# DNAJ Homolog Subfamily C Member 11 Stabilizes SARS-CoV-2 NSP3 to Promote Double-Membrane Vesicle Formation

**DOI:** 10.3390/v17081025

**Published:** 2025-07-22

**Authors:** Shuying Chen, Shanrong Yang, Xiaoning Li, Junqi Xiang, Jiangyu Cai, Yaokai Wang, Qingqing Li, Na Zang, Jiaxu Wang, Jian Shang, Yushun Wan

**Affiliations:** 1 College of Basic Medicine, Chongqing Medical University, Chongqing 400016, China; 18220585379@163.com (S.C.); yshanrong@163.com (S.Y.); lxning0102@163.com (X.L.); 2023130023@stu.cqmu.edu.cn (J.X.); caijiangyu1996@163.com (J.C.); 13864367090@163.com (Y.W.); zangna1214@126.com (N.Z.); 2 Henan Institute of Medical and Pharmaceutical Sciences, Zhengzhou University, Zhengzhou 450052, China; 18039232576@163.com; 3 College of Life Sciences, Henan Normal University, Xinxiang 453000, China; jiaxuw@126.com

**Keywords:** DNAJC11, SARS-CoV-2, viral replication, DMVs

## Abstract

Coronaviruses, particularly those classified as highly pathogenic species, pose a significant threat to global health. These viruses hijack host cellular membranes and proteins to facilitate their replication, primarily through the formation of replication organelles (ROs). However, the precise regulatory mechanisms underlying RO formation remain poorly understood. To elucidate these mechanisms, we conducted mass spectrometry analyses, identifying interactions between the host protein DnaJ homolog subfamily C member 11 (DNAJC11) and the SARS-CoV-2 nonstructural protein 3 (NSP3) protein. Notably, results showed that DNAJC11 depletion reduces SARS-CoV-2 infection, indicating possible positive regulatory involvement. But the ectopic expression of DNAJC11 did not lead to marked alterations in immune or inflammatory responses. DNAJC11 enhanced NSP3 expression stability through endogenous apoptosis pathways and facilitated its interaction with NSP4, thereby promoting the formation of double-membrane vesicles (DMVs). Knockdown of DNAJC11 reduced DMV number and size, accompanied by dysregulation of the endoplasmic reticulum and mitochondria. However, supplementation with DNAJC11 restored both DMV number and size. These findings provide novel insights into the role of DNAJC11 as a host factor that modulates DMV formation and supports SARS-CoV-2 replication by targeting the NSP3 protein. This study advances our understanding of the molecular interactions between host and viral components and highlights DNAJC11 as a potential target for antiviral interventions.

## 1. Introduction

The replication of viruses is intrinsically dependent on the exploitation of host cellular factors and pathways. Proviral host factors represent promising targets for antiviral therapies due to their genetic stability compared to viral components and their potential applicability across related viral species [1]. Despite significant advancements in the development of antiviral drugs and extensive research on coronaviruses (CoVs), the continuous and rapid evolution of these viruses has led to the emergence of drug-resistant variants, complicating antiviral strategies and perpetuating the threat posed by these pathogens [2]. Epidemiological studies have highlighted notable variations in the infectivity of recent novel CoV variants across different populations [3], yet the molecular mechanisms enabling the efficient hijacking of host cellular machinery for viral replication, as well as host defenses against viral replication, remain inadequately understood.

Severe acute respiratory syndrome coronavirus 2 (SARS-CoV-2), a novel beta-coronavirus, shares 79% genome sequence identity with SARS-CoV and 50% identity with Middle East respiratory syndrome coronavirus (MERS-CoV), underscoring its genetic relatedness to these significant public health threats. Beyond its health implications, SARS-CoV-2 also imposes substantial economic burdens [4]. Like other positive-sense RNA viruses, including picornaviruses, arteriviruses, and noroviruses, CoVs rely on the formation of replication organelles (ROs) to sustain their replication cycle [5,6,7]. CoV infections are characterized by the formation of interconnected perinuclear double-membrane structures derived from endoplasmic reticulum (ER), including double-membrane vesicles (DMVs), convoluted membranes, and the recently identified double-membrane spherules [8]. The formation of DMVs, central to the replication of CoVs, is orchestrated by viral nonstructural proteins (NSPs), which play essential roles in membrane remodeling. However, their formation necessitates the involvement of host-derived organelle membranes, particularly from the ER [9]. Among these NSPs, NSP3 and NSP4 are sufficient to induce SARS-CoV-2-mediated DMV formation, whereas NSP6 facilitates the establishment of connections between ER membranes and DMVs [10,11]. Understanding the mechanisms underlying DMV biogenesis and their functional significance in the SARS-CoV-2 replication cycle is pivotal for devising novel antiviral strategies, not only against this pathogen but also for combating other CoVs that may emerge in the future.

We identified DNAJC11, a member of the J protein family and a cofactor of the heat shock protein 40 (Hsp40) family [12], as a critical host factor in SARS-CoV-2 replication. DNAJC11 is defined by a conserved J domain and plays a key role in protein translation, folding, and quality control [13,14]. Although DNAJC11 is highly conserved in both vertebrates and invertebrates, its functional complexity is amplified by the presence of multiple subtypes, and its involvement in regulating viral infections has been minimally explored.

In this study, integrative omic analyses revealed host protein DNAJC11 as a crucial regulator of SARS-CoV-2 replication, specifically through its role in the formation of DMVs. Results showed that DNAJC11 stabilizes the viral protein NSP3 and promotes the interaction of NSP3 and NSP4, essential for DMV biogenesis. These findings provide novel insights into the host-mediated mechanisms underlying DMV formation and identify DNAJC11 as a promising candidate for antiviral intervention.

## 2. Materials and Methods

### 2.1. Cells and Viruses

HEK-293T cells (ATCC, #CRL-3216), Vero-E6 cells (ATCC, #CRL-1586), Calu-3 cells (ATCC, #HTB-55), and HPAEpiCs (BFB, #BFN60807335) were maintained in Dulbecco’s modified Eagle’s medium (DMEM) (Gibco, ThermoFisher Scientific) supplemented with 100 IU/mL penicillin, 100 μg/mL streptomycin, and 10% fetal bovine serum (FBS) (Gibco, ThermoFisher Scientific) at 37 °C in a humidified atmosphere of 5% CO_2_. Calu-3 was kindly provided by Prof. Jincun Zhao (Institute of Infectious Disease, Guangzhou, China). HPAEpiCs were purchased from National Collection of Authenticated Cell Cultures (Shanghai, China) and stored in our laboratory.

HCoV-229E (ATCC, VR-740) was kindly provided by Prof. Jincun Zhao (Institute of Infectious Disease, Guangzhou Eighth People’s Hospital of Guangzhou Medical University, China). VSV-GFP was a kind gift from Prof. Ying Zhu (Wuhan University, China). The RSV A2 and EV-D68 strains were stored in our laboratory. SARS-CoV-2 GFP/∆N VLPs were constructed as previously described [15]. In detail, the full-length SARS-CoV-2 cDNA lacking the N protein was transcribed using a mMESSAGE mMACHINE T7 Transcription Kit (ThermoFisher Scientific, Waltham, MA, USA) in vitro. The SARS-CoV-2 GFP/∆N viral RNA and N mRNA were mixed and co-electroporated into Vero-E6-N cells using the GenePulser apparatus (Bio-Rad, Hercules, CA, USA).

### 2.2. Viral Infection and Amplification Test

Viral titers were estimated using the Reed and Muench method and expressed as the 50% tissue culture infective dose (TCID_50_)/mL, following previously described protocols. The multiplicity of infection (MOI) was calculated based on the virus titer specific to the respective cell lines.

### 2.3. Antibodies and Western Blot Analysis

The antibodies utilized in this study included rabbit anti-DNAJC11 (#DF12948, Affinity Biosciences, Cincinnati, OH, USA), mouse monoclonal anti-GFP (sc-9996, Santa Cruz Biotechnology, Dallas, TX, USA), and rabbit polyclonal anti-Flag (AE092, ABclonal, Wuhan, China), anti-Myc (AE070, ABclonal, Wuhan, China), anti-SARS-CoV-2 NSP8 (A20202, ABclonal, Wuhan, China), and anti-β-actin (AC026, ABclonal, Wuhan, China). A monoclonal antibody against SARS-CoV-2 NSP3 (#88086, Cell Signaling Technology, Danvers, MA, USA) was also used. Secondary antibodies included horseradish peroxidase (HRP)-conjugated goat anti-rabbit (AS014, ABclonal, Wuhan, China), HRP-conjugated goat anti-mouse (AS003, ABclonal, Wuhan, China), FITC-conjugated goat anti-rabbit IgG (H + L) (AS011, ABclonal, Wuhan, China), Cy3-conjugated goat anti-rabbit IgG (H + L) (AS007, ABclonal, Wuhan, China), FITC-conjugated goat anti-mouse IgG (H + L) (AS001, ABclonal, China), ABflo^®^ 647-conjugated goat anti-mouse IgG (H + L) (AS059, ABclonal, Wuhan, China), and Cy3-conjugated goat anti-mouse IgG (H + L) (AS008, ABclonal, Wuhan, China).

For Western blot, whole-cell lysates (WCLs) from treated and untreated cells were generated by adding 1× sodium dodecyl-sulfate polyacrylamide gel electrophoresis (SDS-PAGE) sample buffer to the cells. The proteins were then transferred onto an Immobilon-P membrane (Millipore, Billerica, MA, USA). The membrane was blocked and reacted with appropriate primary antibodies overnight at 4 °C, and then with HRP-conjugated secondary antibodies for 1 h at room temperature. The antibody–antigen complexes were visualized using enhanced chemiluminescence detection reagents (Thermo, Waltham, MA, USA) and quantified with ImageJ software version 1.54 (National Institutes of Health, Bethesda, MD, USA).

### 2.4. Plasmid Construction and Cell Transfection

Each full-length viral coding region fragment of SARS-CoV-2 was cloned and inserted into the pcDNA3.1-3xFlag vector to establish eukaryotic plasmids expressing Flag-tagged viral proteins. Plasmids encoding GFP-tagged SARS-CoV-2 NSP3 and mCherry-tagged SARS-CoV-2 NSP4 were constructed in our laboratory. A series of SARS-CoV-2-truncated NSP3 mutants and DNAJC11 mutants were generated via conventional polymerase chain reaction (PCR), with the GFP-NSP3 and pcDNA3.1 vector plasmids used as templates. Plasmids encoding Myc-tagged DNAJC11 and its truncated mutants were constructed using the pcDNA3.1-Myc-His(−) vector (Addgene, Watertown, MA, USA). All plasmid sequences were confirmed via Sanger sequencing.

The cells were transfected with various plasmids using Hieff Trans^TM^ Liposomal Transfection Reagent (Yeasen, Shanghai, China) according to the manufacturer’s protocols. Briefly, for all plasmid transfections, HEK293T or Calu-3 cells were seeded on the indicated cell culture dishes before transfection. When cell confluence reached approximately 70%, the medium was replaced with fresh complete medium, and the transfection mixture was prepared at a plasmid-to-liposome ratio of 1:2. The mixture was added to the cell cultures after 15 min. After 24 h of transfection, the cells were harvested for different experiments.

### 2.5. Immunofluorescence and Confocal Immunofluorescence Microscopy

Cells were grown on Nunc glass-bottom dishes to 70% confluency and infected with SARS-CoV-2-VLPs at an MOI of 1. The cells were then washed three times with phosphate-buffered saline (PBS) and fixed in 4% paraformaldehyde in PBS at 4 °C for 2 h. The cells were again washed with PBS three times and treated with 0.3% Triton X-100 for 15 min. Briefly, 5% bovine serum albumin (BSA) was used as a blocking buffer for 2 h at room temperature, after which the cells were incubated with appropriate primary antibodies overnight at 4 °C, then washed and incubated with the indicated secondary antibodies. Nuclei were stained with 4′,6-diamidino-2-phenylindole (DAPI, (Beyotime Biotechnology, Shanghai, China) at a dilution of 1 μg/mL for 5 min, then examined via confocal microscopy (LSCM, Leica SP8, Solms, Germany).

### 2.6. Co-Immunoprecipitation (Co-IP) Analysis

HEK293T cells were grown in 6 cm dishes, and the cellular monolayer was cotransfected with indicated plasmids. The transfected cells were cultured and lysed in 0.5 mL of lysis buffer (20 mM Tris pH 7.5, 150 mM NaCl, 1% Triton X-100, 1 mM EDTA, 10 µg/mL aprotinin, 10 µg/mL leupeptin, and 1 mM PMSF). For each sample, 0.5 mL of lysate was incubated with 0.5 mg of suitable antibody and 30 μL of protein A/G-Sepharose (Santa Cruz, CA, USA) for 12 h. The Sepharose beads were washed three times with 1 mL of lysis buffer containing 500 mM NaCl. The precipitates were analyzed by immunoblotting.

### 2.7. Protein Expression and Purification

Human DNAJC11 (UniProt ID: Q9NVH1) cDNA was cloned and inserted into a pcDNA3.1-Myc-6His vector with an N-terminal His tag. Subsequently, 2 mg of plasmid and 4 mg of polyethylenimine (Yeasen) were preincubated in 50 mL of fresh Union 293 medium (Union-Biotech) for 30 min before being added to 1 L of HEK293F cells at a density of 2.0 × 10^6^ cells/mL. After 48 h of incubation at 37 °C under 5% CO_2_, the cells were harvested and resuspended in buffer containing 25 mM HEPES (pH 7.4), 150 mM NaCl, 5 μg/mL aprotinin, 1 μg/mL pepstatin, and 5 μg/mL leupeptin. The cell membrane was solubilized with 2% (*w*/*v*) n-dodecyl-β-D-maltoside (DDM) at 4 °C for 2 h. After high-speed centrifugation at 20,000× *g* for 30 min at 4 °C, the supernatant was loaded onto anti-His resin (Cytiva). The eluate was subjected to a Superdex 200 gel filtration column (GE Healthcare, Chicago, IL, USA) and peak fractions were pooled for further experiments.

### 2.8. Pull-Down Assay

A total of 2 µg of 6 × His-tagged DNAJC11 was co-incubated overnight at 4 °C with GFP-immobilized NSP3 in 600 μL of lysis buffer containing 150 mM NaCl, 50 mM Tris-HCl (pH 7.5), 5 mM DTT, 0.1% NP-40, and 0.25 mg/mL BSA, under continuous rotation. The beads were then washed five times with PBST, followed by boiling with SDS loading buffer for 10 min. The resulting samples were analyzed by Western blot.

### 2.9. Quantitative Real-Time PCR (qRT-PCR)

Total RNA was extracted from the treated cells using TRIzol reagent (Invitrogen, Carlsbad, CA, USA) according to the manufacturer’s instructions, then reverse transcribed into cDNA using a RevertAid First-Strand cDNA Synthesis Kit (ABclonal) according to the manufacturer’s protocols. qRT-PCR was performed with SYBR Premix ExTaq reagents (ABclonal) to quantify the abundance of various RNAs. The number of cycles required for target gene amplification was obtained, and the relative expression of the target gene was calculated using the comparative cycle threshold (2^−ΔΔCT^) method. The primers used for qRT-PCR are listed in Supplementary Table S1.

### 2.10. Lentivirus-Mediated Stable Cell Line Construction

HEK293T cells were grown to 60% confluence. The lentiviral packaging plasmid and DNAJC11 shRNA were cotransfected into the cells at a specific ratio (psPAX2:pMD2.G:shRNA = 0.75:0.25:1) through transfection. The medium was replaced with fresh DMEM after 6 h of transfection. After 48 h of transfection, the culture medium was collected and centrifuged at 4000 rpm for 5 min at 4 °C to obtain the virus mixture. The receptor cells were infected with the lentivirus in the presence of polybrene (8 μg/mL). The cells were selected at 48 h post-infection and maintained in 10% FBS-DMEM supplemented with puromycin. DNAJC11 knockdown efficiency was detected using qRT-PCR or immunoblotting with anti-DNAJC11 antibodies. The DNAJC11 shRNA sequence used was 5′-CCGGGGGAGTCTACAAATTTGGAAACTCGAGTTTCCAAATTTGTAGACTCCCTTTTTG-3′. The scrambled shRNA sequence used was 5′-CCGGCAACAAGATGAAGAGCACCAACTCGAGTTGGTGCTCTTCATCTTGTTGTTTTTG-3′.

### 2.11. Transmission Electron Microscopy (TEM)

Calu-3-N cells were washed three times (15 min each) with 0.1 M phosphate buffer, fixed in 2% aqueous osmium tetroxide for 1 h, and washed three times (15 min each) with deionized water. The samples were then dyed with 2% uranyl acetate for 30 min and dehydrated through graded alcohols (50–100%) and 100% acetone for 15 min each. After that, the samples were embedded in EPON 812 resin and cured for 24 h at 37 °C, 45 °C, or 60 °C. Ultrathin (70 nm) sections were obtained using an ultrathin slicer and stained with 2% uranyl acetate and 0.3% lead citrate. Images of the samples were taken using a Tecnai G2 Spirit transmission electron microscope (FEI Company, Hillsboro, OR, USA). Representative images of at least three independent replicate experiments are shown.

### 2.12. Cell Viability Assay

Cell viability was assessed using the Cell Counting Kit-8 (CCK-8) (Abbkine, BMU106, Wuhan, China). Cells were either transfected with the corresponding plasmids or pretreated with specific inhibitors, followed by the addition of CCK-8 reagent to each well. After incubation at 37 °C for 30 min, absorbance at 450 nm was measured using a Tecan Spark^®^ multimode microplate reader (Tecan Group Ltd., Männedorf, Switzerland).

The following inhibitors were purchased from MedChemExpress (MCE, Monmouth Junction, NJ, USA): cycloheximide (CHX, Catalog No. HY-12320, purity ≥ 99%), KNK437 (Catalog No. HY-100110, purity ≥ 98%), MG132 (Catalog No. HY-13259, purity ≥ 99%), bafilomycin A1 (Baf-A1, Catalog No. HY-100558, purity ≥ 99%), chloroquine (CQ, Catalog No. HY-17589A, purity ≥ 99%), Z-VAD-FMK (Catalog No. HY-16658B, purity ≥ 99%), Z-IETD-FMK (Catalog No. HY-101297, purity ≥ 98%), and ABT-737 (Catalog No. HY-50907, purity ≥ 99%). Stock solutions were prepared in DMSO and stored at −80 °C.

### 2.13. Quantification and Statistical Analysis

All data are presented as means ± standard deviations (SDs) of three independent experiments. Two-tailed Student’s *t*-test was used to analyze the significance of the data. Differences were considered significant at * *p* < 0.05 and highly significant at ** *p* < 0.01.

## 3. Results

### 3.1. DNAJC11 Facilitates SARS-CoV-2 Replication

**Figure 1:**
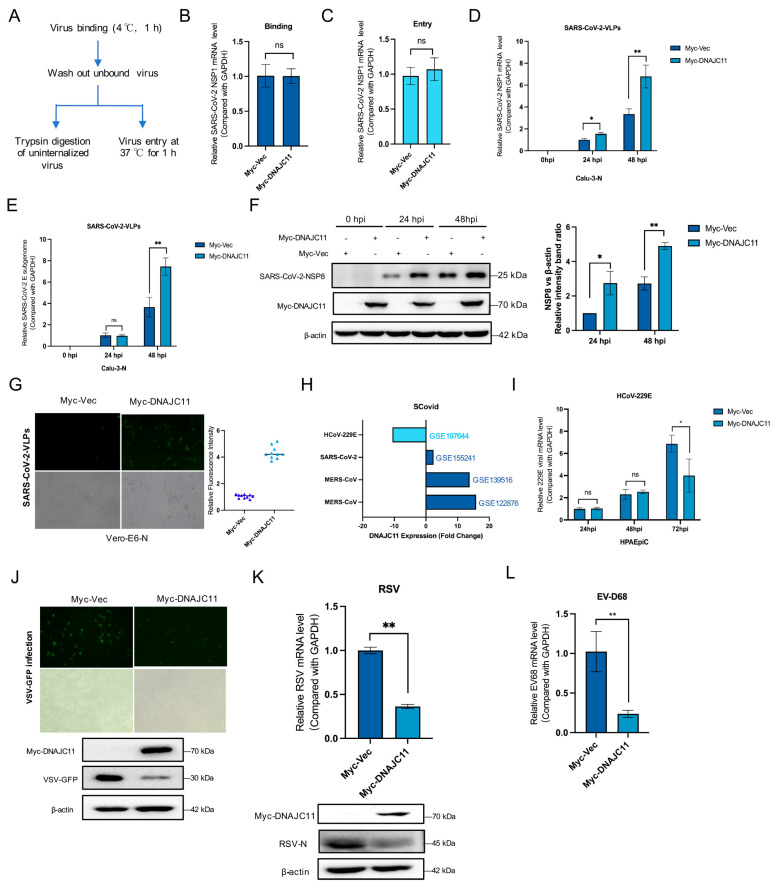
DNAJC11 overexpression promotes SARS-CoV-2 replication. (**A**) Schematic of virus binding and entry assay in Myc-Vec or Myc-DNAJC11-overexpressed cells. Amount of viral RNA in Myc-Vec-overexpressed cells was normalized to 1. (**B**) Calu-3 cells were pre-transfected with vector or Myc-DNAJC11 for 24 h, then infected with SARS-CoV-2-VLPs (MOI = 5) at 4 °C for 1 h. mRNA levels of SARS-CoV-2 were measured by qRT-PCR. (**C**) Calu-3 cells were infected with SARS-CoV-2-VLPs (MOI = 5) at 4 °C for 1 h, then shifted to 37 °C for up to 1 h. qRT-qPCR analysis was used to detect the mRNA expression level of SARS-CoV-2. (**D**,**E**) Calu-3-N cells were transfected with vector or Myc-DNAJC11 for 24 h, then infected with SARS-CoV-2-VLPs for 0, 24, and 48 h. qRT-PCR was used to detect the NSP1 (**D**) and E mRNA expression levels (**E**) in SARS-CoV-2. (**F**) Calu-3-N cells were transfected with either vector or Myc-DNAJC11 for 24 h, followed by infection with SARS-CoV-2-VLPs for 0, 24, and 48 h. Cells were lysed for Western blot, and target protein levels relative to the β-actin levels in the DNAJC11-overexpressing and Vec-transfected cells were normalized using Image J. (**G**) Vero-E6-N cells were transfected with vector or Myc-DNAJC11 for 24 h, then infected with SARS-CoV-2-VLPs for 48 h. GFP fluorescence was observed via fluorescence microscopy. Fluorescence intensity was calculated using ImageJ software. (**H**) Expression level of DNAJC11 was retrieved from the SCovid database. (**I**) HPAEpiCs were transfected with vector or Myc-DNAJC11 for 24 h, then infected with HCoV-229E for 24, 48, and 72 h. mRNA levels of HCoV-229E were quantified using qRT-PCR. (**J**) Vero cells were transfected with vector or Myc-DNAJC11 for 24 h, then infected with VSV-GFP for 24 h. GFP was observed by immunofluorescence assay and Western blot. (**K**) HPAEpiCs were transfected with vector or Myc-DNAJC11 for 24 h, then infected with RSV-A2 for 24 h. mRNA and protein levels of RSV were detected by qRT-PCR and Western blot. (**L**) RD cells were transfected with vector or Myc-DNAJC11 for 24 h, then infected with EV-D68 for 24 h. mRNA levels of EV68 were detected by qRT-PCR. Data are presented as mean ± SD of three independent experiments. Statistical significance was analyzed by Student’s *t*-test (* *p* < 0.05, ** *p* < 0.01, ns represents no significance).

Large-scale functional genetic and interactome analyses, including studies of protein interactions induced by overexpression of viral proteins in uninfected cells, have been extensively employed in CoV research to identify many previously unknown pro- and antiviral host factors [16,17,18]. Comparative analysis of several omics studies highlighted DNAJC11 as a potential host factor (Figure S1A). DNAJC11, a component of the mitochondrial outer membrane, is recognized for its involvement in membrane remodeling and organelle dynamics. To investigate its role, affinity purification-mass spectrometry (AP-MS) was performed in HEK-293T cells co-expressing His-tagged NSP3 and Flag-tagged NSP4 proteins. Following copurification using Ni columns and molecular sieves, MS analysis identified DNAJC11 as a protein potentially involved in SARS-CoV-2 infection (Figure S1B–D). Additionally, our mass spectrometry analysis identified key proteins linked to DMV formation, such as TRAM1, FXR1, and RTN4. The identification of these proteins further strengthens confidence in the reliability of our results. SARS-CoV-2 transcription and replication-competent virus-like-particles (trVLP) express a reporter gene (EGFP), replacing viral nucleocapsid gene (N), retaining the ability to complete the viral replication cycle exclusively in cells expressing exogenous SARS-CoV or SARS-CoV-2 N protein [15]. This study employed Calu-3 cells to establish a trans-complementation replicon system to support trVLP propagation. In brief, Calu-3 cells supplemented with SARS-CoV-2 N protein (Calu-3-N) were infected with SARS-CoV-2-GFP/∆N virus-like particles (VLPs) for 48 h. The role of DNAJC11 in viral binding and entry was examined using Calu-3-N cells overexpressing Myc-Vec- and Myc-DNAJC11 (Figure 1A). Cells were infected with SARS-CoV-2 VLPs, and viral binding and entry were quantified via qRT-PCR. Results revealed that neither the binding (Figure 1B) nor entry (Figure 1C) of SARS-CoV-2-VLPs changed significantly when cells overexpressed DNAJC11.

The ability of DNAJC11 to influence viral replication was subsequently analyzed. Calu-3-N cells were transfected with Myc-Vec or Myc-DNAJC11 for 24 h, then infected with SARS-CoV-2-VLPs. Viral mRNA (NSP1, E) and protein expression levels (NSP8) were assessed at 0, 24, or 48 h post-infection using qRT-PCR and Western blot. Results showed that overexpression of DNAJC11 significantly enhanced viral replication (Figure 1D–F). Cell viability was also assessed at indicated time points following DNAJC11 overexpression, confirming that the observed enhancement of viral replication was not attributable to changes in cellular health (Figure S2A). Vero cells, derived from African green monkey kidney cells, lack endogenous interferon production and possess attenuated innate immune signaling, making them an ideal model for studying viral replication dynamics independent of host interferon responses [19]. In this context, Vero-E6 cells supplemented with SARS-CoV-2 N protein (Vero-E6-N) were infected with SARS-CoV-2-EGFP/∆N VLPs for 48 h. Fluorescence microscopy observation revealed higher viral signal intensity in Myc-DNAJC11-transfected cells compared to Myc-Vec-expressing cells, further supporting its role in facilitating SARS-CoV-2 replication (Figure 1G).

Differential expression of DNAJC11 during CoV infections was analyzed using the SCovid database (http://bio-computing.hrbmu.edu.cn/scovid/#/home, accessed on 1 October 2024). Elevated DNAJC11 expression was detected in infections caused by highly pathogenic CoVs, such as MERS-CoV and SARS-CoV-2, while significantly lower expression was observed in HCoV-229E infections (Figure 1H). Building on these interesting findings, the involvement of DNAJC11 in other CoV infections was investigated by transfecting human type II alveolar epithelial cells (HPAEpiCs) with either Myc-Vec or Myc-DNAJC11, followed by infection with HCoV-229E. Viral mRNA was analyzed at 24, 48, or 72 h post-infection using qRT-PCR. Interestingly, overexpression of DNAJC11 reduced replication levels of HCoV-229E at the late stage of viral replication (Figure 1I). Cytotoxicity assays confirmed that overexpression of Myc-Vec or Myc- DNAJC11 in HPAEpiCs cells for 24, 48, and 72 h did not impair cell viability (Figure S2B). Further analysis of other RNA viruses, such as vesicular stomatitis virus (VSV-GFP) (Figure 1J), respiratory syncytial virus (RSV-A2) (Figure 1K), and enterovirus-D68 (EV-D68) (Figure 1L), indicated that DNAJC11 overexpression significantly reduced replication, as determined by immunofluorescence, qRT-PCR, and Western blot.

**Figure 2:**
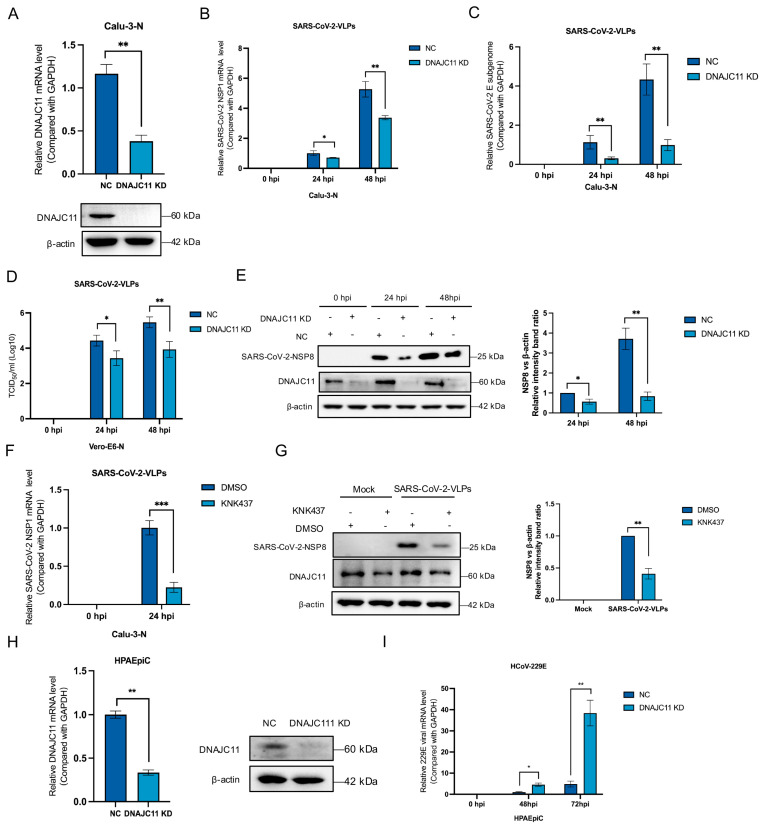
Knockdown of DNAJC11 inhibits SARS-CoV-2 replication. (**A**) mRNA and protein levels of DNAJC11 were detected in NC and KD Calu-3-N cells via qRT-PCR and Western blot. (**B**–**D**) NC or KD Calu-3-N cells were infected with SARS-CoV-2-VLPs for 0, 24, and 48 h. Viral replication was measured by qRT-PCR (**B**,**C**), TCID50 (**D**), and Western blot (**E**). The NSP8 expression levels relative to the β-actin levels in the DNAJC11 KD and NC-transfected cells were normalized using Image J. (**F**,**G**) Calu-3-N cells were pretreated with 100 µM KNK437 for 1 h, then infected with SARS-CoV-2-VLPs (MOI = 1) at 24 h post-infection (hpi). Viral replication was measured by qRT-PCR (**F**) and Western blot (**G**). The NSP8 expression levels relative to the β-actin levels in the DMSO and KNK437-treated cells were normalized using Image J. (**H**) mRNA and protein levels of DNAJC11 were detected in NC and KD HPAEpiCs via qRT-PCR and Western blot. (**I**) DNAJC11 NC and KD HPAEpiCs were infected with HCoV-229E for 0, 48, and 72 h. HCoV-229E mRNA level was then detected via qRT-PCR. Data are presented as mean ± SD of three independent experiments. Statistical significance was analyzed by Student’s *t*-test (* *p* < 0.05, ** *p* < 0.01, *** *p* < 0.001).

To further elucidate the role of DNAJC11 in SARS-CoV-2 replication, stable DNAJC11-knockdown (KD) cell lines were established using a lentivirus packaging system. Knockdown efficiency was achieved by transfecting cells with DNAJC11-specific short hairpin RNA (shRNA) (Figure 2A), leading to a substantial reduction in DNAJC11 expression. Control cells transfected with pLKO-scramble RNA (NC) and DNAJC11-KD cells were either mock-infected or infected with SARS-CoV-2-VLPs for 0, 24, or 48 h. Subsequent qRT-PCR analysis of SARS-CoV-2 replication revealed a significant reduction in viral NSP1 (Figure 2B) and E (Figure 2C) mRNA levels in the DNAJC11-KD cells compared to the NC cells. To assess the impact on viral production, virus yields from SARS-CoV-2-infected NC or DNAJC11-KD cells were detected using a TCID_50_ assay. A significant decrease in viral titers was observed in DNAJC11-KD cells compared to the control group (Figure 2D). Western blot analysis provided further evidence of reduced SARS-CoV-2 replication in DNAJC11-KD cells. Both NC and DNAJC11-KD cells were either mock-infected or infected with SARS-CoV-2-VLPs for 0, 24, or 48 h. The expression levels of the SARS-CoV-2 NSP8 protein, a marker of viral replication, and DNAJC11 were assessed using corresponding antibodies. Results demonstrated a substantial reduction in NSP8 expression in DNAJC11-KD cells compared to NC cells, indicating a significant decrease in SARS-CoV-2 replication (Figure 2E). The cytotoxicity assay showed that DNAJC11 knockdown in Calu-3-N cells did not affect cell viability (Figure S2C).

Given the established role of DNAJC11 as a cofactor of the Hsp40 family, its involvement in numerous biological processes was further explored in the context of SARS-CoV-2 infection. To investigate the potential contribution of Hsps to viral replication, Hsp synthesis was inhibited in Calu-3-N cells using KNK437, a selective inhibitor of Hsp synthesis in vitro. Following inhibition, the cells were infected with SARS-CoV-2-VLPs, and viral replication was detected by qRT-PCR and Western blot.

Treatment with KNK437 significantly suppressed SARS-CoV-2 replication, as demonstrated by a marked reduction in viral NSP1 mRNA levels compared to DMSO-treated controls (Figure 2F). To further evaluate viral replication, the expression of the SARS-CoV-2 NSP8 protein was detected. Western blot analysis confirmed that KNK437 effectively down-regulated SARS-CoV-2 replication (Figure 2G). Similarly, DNAJC11 expression was knocked down in HPAEpiCs (Figure 2H), and the replication of HCoV-229E in DNAJC11-KD cells was quantified using qRT-PCR. Compared to NC cells, the viral mRNA levels of HCoV-229E were significantly increased in the DNAJC11-KD HPAEpiCs (Figure 2I). The cytotoxicity test also demonstrated that DNAJC11 knockdown in HPAEpiCs for 0, 48, and 72 h did not affect cell viability (Figure S2D). Collectively, these findings highlight the specific role of DNAJC11 in supporting SARS-CoV-2 replication.

### 3.2. DNAJC11 Does Not Promote SARS-CoV-2 Infection Through Type I IFN or IFN-Stimulated Gene (ISG) Pathways

**Figure 3:**
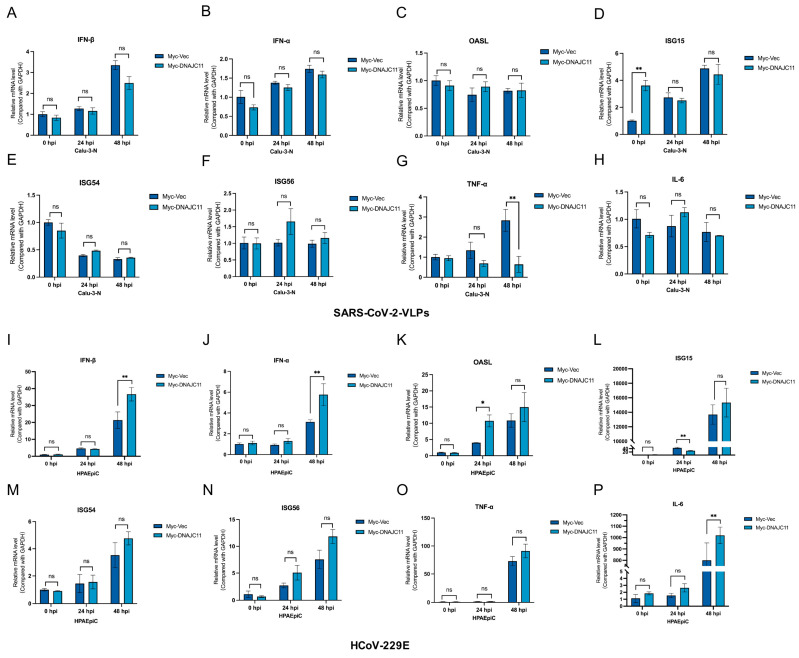
DNAJC11 does not affect type I IFN production or ISG expression. (**A**–**H**) Calu-3-N cells were transfected with vector or Myc-DNAJC11 for 24 h, then infected with SARS-CoV-2-VLPs for 0, 24, and 48 h. mRNA levels of IFN-β (**A**), IFN-α (**B**), OASL (**C**), ISG15 (**D**), ISG54 (**E**), ISG56 (**F**), TNF-α (**G**), and IL-6 (**H**) were quantified using qRT-PCR. (**I**–**P**) HPAEpiCs were transfected with vector or Myc-DNAJC11 for 24 h, then infected with HCoV-229E for 0, 24, and 48 h. mRNA levels of IFN-β (**I**), IFN-α (**J**), OASL (**K**), ISG15 (**L**), ISG54 (**M**), ISG56 (**N**), TNF-α (**O**), and IL-6 (**P**) were quantified using qRT-PCR (* *p* < 0.05, ** *p* < 0.01, ns represents no significance).

The host response to RNA viral infection is critically dependent on the activation of pattern recognition receptors (PRRs), which initiate signaling cascades leading to the production of pro-inflammatory cytokines, type I and type III IFNs, and ISGs [20]. To determine whether DNAJC11 influences these innate immune and inflammatory responses during SARS-CoV-2 infection, Calu-3-N cells were transfected with Myc-Vec or Myc-DNAJC11-expressing plasmids and subsequently infected with SARS-CoV-2-VLPs for 0, 24, or 48 h. The mRNA expression levels of IFN-β, IFN-α, OASL, ISG15, ISG54, ISG56, TNF-α, and IL-6 were quantified via qRT-PCR. Consistently, overexpression of DNAJC11 did not significantly alter the expression of these innate immune response-related genes (IFN-β, IFN-α, OASL, ISG15, ISG54, and ISG56) and inflammatory response-related genes (TNF-α and IL-6) (Figure 3A–H).

The role of DNAJC11 in modulating the innate immune response during other coronavirus infections was also investigated. DNAJC11-overexpressing HPAEpiCs were infected with HCoV-229E, and gene expression was analyzed. Interestingly, overexpression of DNAJC11 enhanced HCoV-229E-induced up-regulation of IFN-β, IFN-α, and IL-6 but did not significantly affect the expression of TNF-α, OASL, ISG15, ISG54, or ISG56 (Figure 3I–P). Previous studies have shown that DNAJC11 overexpression inhibits the replication of other RNA viruses (Figure 1), suggesting that the innate immune response and inflammatory pathways may mediate its antiviral effects. To test this, DNAJC11 was overexpressed in HPAEpiCs prior to VSV infection. While IFN-α expression was significantly increased, no notable changes were observed in the mRNA levels of IFN-β, ISG15, TNF-α, or IL-6 (Figure S3A–E). Similarly, in HPAEpiCs transfected with Myc-Vec or Myc-DNAJC11 and subsequently infected with RSV-A2 for 48 h, a marked increase in the mRNA levels of IFN-β, IFN-α, TNF-α, and IL-6 was observed compared to Vec-transfected cells (Figure S3F–J). Collectively, these findings indicate that the pro-replicative function of DNAJC11 in SARS-CoV-2 infection is not contingent upon the innate immune response or inflammatory signaling pathways.

### 3.3. SARS-CoV-2 NSP3 Interacts with DNAJC11

**Figure 4:**
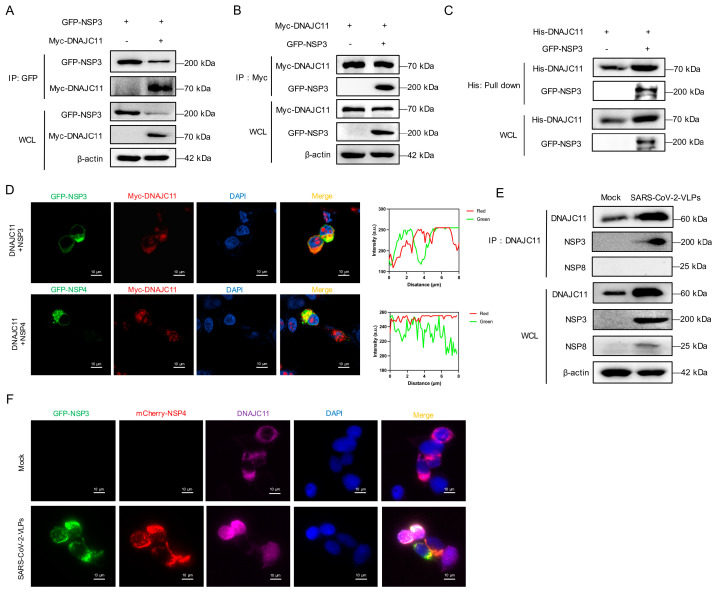
DNAJC11 interacts with SARS-CoV-2 NSP3. (**A**,**B**) HEK293T cells were cotransfected with vector or GFP-NSP3 and Myc-DNAJC11 for 24 h. Cells were lysed and immunoprecipitated with anti-Myc or anti-GFP antibodies. WCLs and IP complexes were analyzed by Western blot. (**C**) HEK293T cells were transfected with vector or GFP-NSP3 for 24 h. Cell lysates were harvested and subjected to a pull-down assay with purified His-tagged DNAJC11 protein. Pull-down samples were analyzed via Western blot with anti-His and anti-GFP antibodies. (**D**) HEK293T cells were cotransfected with indicated plasmids for 24 h. Subcellular locations of GFP-tagged NSP3, NSP4 (green), Myc-tagged DNAJC11 (red), and nuclear marker DAPI (blue) were analyzed via confocal microscopy. Scale bar: 0.1 μm. (**E**) Calu-3-N cells were mock-infected or infected with SARS-CoV-2-VLPs for 48 h. Cells were lysed and immunoprecipitated with indicated antibodies. WCL and IP complexes were detected by anti-NSP3, anti-NSP8, anti-DNAJC11, or anti-β-actin antibodies for Western blot. (**F**) Calu-3-N cells were cotransfected with indicated plasmids for 24 h, then infected with SARS-CoV-2-VLPs for 24 h. Subcellular locations of GFP-tagged NSP3 (green), mCherry-tagged NSP4 (red), endogenous DNAJC11 (purple), and nuclear marker DAPI (blue) were analyzed via confocal microscopy. Scale bar: 0.1 μm.

To explore the mechanism by which DNAJC11 regulates SARS-CoV-2 replication, co-immunoprecipitation (co-IP) and immunofluorescence assays were performed to determine the interaction between the DNAJC11 and SARS-CoV-2 viral proteins. Co-IP analysis confirmed that DNAJC11 specifically interacted with the SARS-CoV-2 NSP3 (Figure 4A,B). To verify whether there was a direct interaction between DNAJC11 and NSP3, His-tagged DNAJC11 protein was purified from 293F cells using Ni-affinity chromatography, followed by a pull-down assay. Results revealed that DNAJC11 directly interacted with GFP-tagged NSP3 (Figure 4C). Additional co-IP experiments were performed to evaluate whether DNAJC11 interacts with other SARS-CoV-2 proteins. Flag-tagged SARS-CoV-2 proteins and Myc-tagged DNAJC11 were co-expressed in 293T cells. The assays revealed that DNAJC11 did not interact with other SARS-CoV-2 proteins, except for the nucleocapsid (N) protein (Figure S4).

Given the established roles of NSP3 and NSP4 in DMV biogenesis, their localization relative to DNAJC11 was examined. Immunofluorescence analysis indicated that both NSP3 and NSP4 colocalized with DNAJC11 in the cytoplasm, forming distinct spot-like distributions surrounding the nucleus (Figure 4D). The interaction between endogenous DNAJC11 and NSP3 in SARS-CoV-2-infected cells was explored. Co-IP analysis revealed that endogenous DNAJC11 clearly co-precipitated with SARS-CoV-2 NSP3 but not with NSP8, which acted as a negative control (Figure 4E). Immunofluorescence analysis of SARS-CoV-2-VLP-infected cells further confirmed the interaction. Endogenous DNAJC11 was primarily localized in the cytoplasm, where it colocalized with GFP-NSP3 and mCherry-NSP4 in spot-like distributions (Figure 4F). Given the divergent effects of DNAJC11 on SARS-CoV-2 and HCoV-229E replication, the interaction between DNAJC11 and the NSP3 protein of HCoV-229E was assessed. Co-IP analysis revealed no detectable binding between DNAJC11 and HCoV-229E NSP3 (Figure S5A). These results confirmed that DNAJC11 specifically associates with SARS-CoV-2 NSP3, consistent with its role in promoting SARS-CoV-2 replication.

### 3.4. DNAJC11 J Domain Interacts with SARS-CoV-2 NSP3

**Figure 5:**
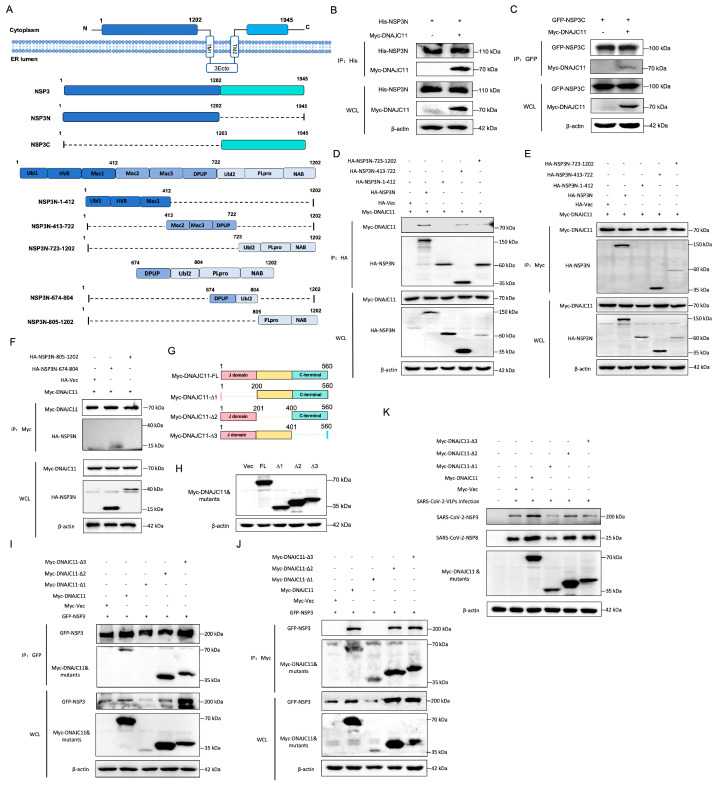
DNAJC11 J domain interacts with SARS-CoV-2 NSP3. (**A**) Schematic of NSP3 and its mutant constructs. (**B**,**C**) HEK293T cells were cotransfected with vector or His-NSP3N and Myc-DNAJC11 (**B**) or GFP-NSP3C and Myc-DNAJC11 (**C**) for 24 h. Cells were lysed and immunoprecipitated with anti-His or anti-GFP antibodies. WCLs and IP complexes were analyzed by Western blot with indicated antibodies. (**D**,**E**) HEK293T cells were cotransfected with Myc-DNAJC11, HA-vector, or HA-tagged truncated NSP3N mutant-expressing plasmids for 24 h. Cell lysates were immunoprecipitated with anti-HA (**D**) or anti-Myc (**E**) antibodies. (**F**) HEK293T cells were cotransfected with Myc-DNAJC11, HA-vector, or HA-tagged truncated NSP3N mutant-expressing plasmids for 24 h. Cells were lysed and immunoprecipitated with anti-His antibodies. WCLs and IP complexes were analyzed by Western blot with indicated antibodies. (**G**) Schematic of Myc-tagged truncated DNAJC11 constructs. (**H**) Expression of Myc-tagged DNAJC11 and mutant proteins was validated by Western blot. (**I**,**J**) HEK293T cells were cotransfected with GFP-NSP3, Myc vector, Myc-DNAJC11, or Myc-tagged truncated DNAJC11 mutant-expressing plasmids for 24 h. Cell lysates were immunoprecipitated with anti-GFP (**I**) or anti-Myc (**J**) antibodies. WCLs and IP complexes were then analyzed by Western blot using indicated antibodies. (**K**) Calu-3-N cells were cotransfected with Myc vector, Myc-DNAJC11, or Myc-tagged truncated DNAJC11 mutant-expressing plasmids for 24 h, then infected with SARS-CoV-2-VLPs (MOI = 1) for 24 h. Cell lysates were detected using Western blot with indicated antibodies.

The SARS-CoV-2 NSP3 protein comprises 1945 amino acid residues organized into at least 15 functional domains, including ubiquitin-like, acidic, macrodomains, papain-like protease (PLpro), and transmembrane domains, providing numerous potentially druggable sites within a single polypeptide. To investigate the interaction between NSP3 and DNAJC11, plasmids expressing the NSP3 N-terminal domain (His-NSP3N) or C-terminal domain (GFP-NSP3C) were constructed, as shown in Figure 5A. The co-IP assays demonstrated that NSP3N (Figure 5B) but not NSP3C (Figure 5C) interacted with DNAJC11. To delineate the specific region of NSP3 responsible for interacting with DNAJC11, the NSP3N fragment was segmented into three structured domains: Ubl1-HVR-Mac1 (aa 1–412), Mac2-Mac3-DPUP (aa 413–722), and Ubl2-PLpro-NAB (aa 723–1202). HA-tagged truncation constructs corresponding to each region were generated and subjected to Co-IP analysis. Notably, DNAJC11 exhibited no interaction with the Ubl1-HVR-Mac1 domain but clearly associated with both the Mac2-Mac3-DPUP and Ubl2-PLpro-NAB domains (Figure 5D,E). To refine the binding interface, a construct encoding the DPUP-Ubl2 region (aa 674–804) was generated. Co-IP analysis confirmed that DNAJC11 selectively interacted with the DPUP-Ubl2 subdomain of NSP3 (Figure 5F).

DNAJC11 belongs to the DNAJ family, characterized by the presence of an N-terminal J domain. This domain, with a flexible ring structure, is necessary for triggering Hsp70 hydrolysis, while the C-terminal domain primarily participates in protein–protein interactions [21]. To identify the key regions of DNAJC11 involved in the interaction with NSP3, a series of truncated DNAJC11 mutants were generated based on the structural features of the protein (Figure 5G). Expression of these mutants was confirmed by Western blot analysis (Figure 5H). The interaction between NSP3 and various DNAJC11 mutants was further evaluated using co-IP assays. Deletion of aa 201 to 400 and 401 to 560 in DNAJC11 had no significant effect on the interaction. However, deletion of aa 1 to 200 completely abrogated the NSP3-DNAJC11 interaction (Figure 5I,J). To assess the functional consequences of this deletion, the replication of SARS-CoV-2 was evaluated in cells expressing the DNAJC11 mutant lacking aa 1 to 200. Western blotting revealed a significant decrease in viral replication in these cells (Figure 5K). These findings highlight the critical role of the N-terminal 1–200 region of DNAJC11 in its interaction with SARS-CoV-2 NSP3, which is essential for supporting viral replication. This region represents a potential target for inhibiting SARS-CoV-2 infection.

### 3.5. DNAJC11 Facilitates DMV Formation

**Figure 6:**
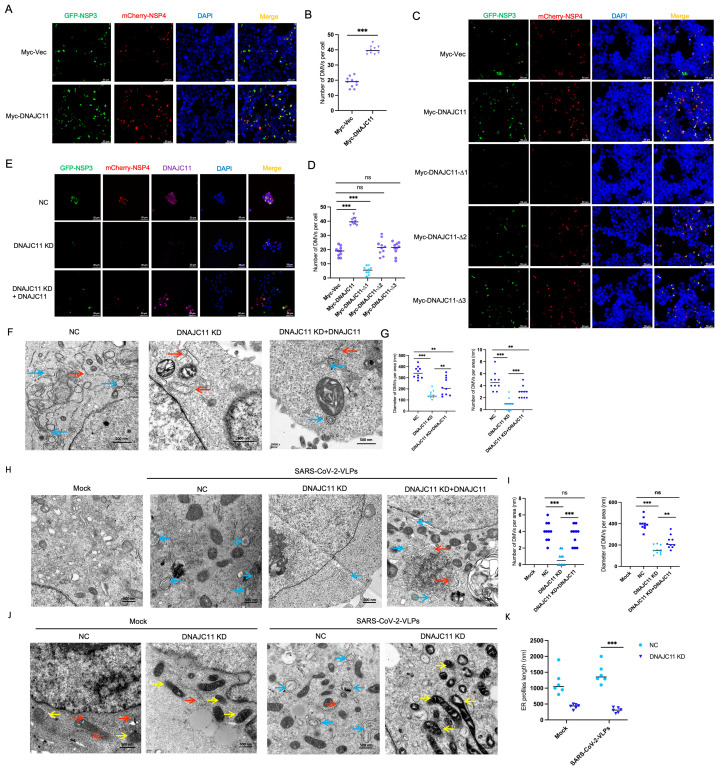
DNAJC11 increases DMV formation and changes ER morphology. (**A**) Calu-3 cells were cotransfected with GFP-NSP3, mCherry-NSP4, and empty vector or Myc-DNAJC11 plasmids for 24 h. Representative fluorescence images were obtained via fluorescence microscopy. Scale bar: 0.5 μm. (**B**) Quantification of DMV number in vector- or Myc-DNAJC11-overexpressing cells. N > 10 fields for each cell line from three independent experiments. Scale bar: 0.5 μm. (**C**) Calu-3 cells were cotransfected with GFP-NSP3, mCherry-NSP4, and empty vector or Myc-DNAJC11 or Myc-tagged truncated DNAJC11 mutant-expressing plasmids. Representative fluorescence images were observed via fluorescence microscopy. Scale bar: 0.5 μm. (**D**) Quantitative analysis of DMV number in panel C. (**E**) DNAJC11 NC-, KD-, or KD-supplemented DNAJC11 cells were cotransfected with GFP-NSP3 or mCherry-NSP4 for 48 h, then fixed and immunostained with anti-DNAJC11 for immunofluorescence assay. (**F**) DNAJC11 NC, KD, or KD + DNAJC11 cells were cotransfected with GFP-NSP3 or mCherry-NSP4 for 48 h, then subjected to TEM. Blue arrows indicate DMVs, red arrows indicate ER profiles. Scale bar: 0.5 μm; amplification: 10,000-fold. (**G**) Average number and diameter of DMVs measured on EM images. Unpaired two-tailed *t*-tests were used. Differences were considered significant at ** *p* < 0.01, *** *p* < 0.001. (**H**) NC, DNAJC11 KD, or KD + DNAJC11 Calu-3-N cells were mock-infected or infected with SARS-CoV-2-VLPs for 48 h, then subjected to TEM. Blue arrows indicate DMVs, red arrows indicate ER profiles. Scale bar: 0.5 μm; amplification: 10,000-fold. (**I**) Average number and diameter of DMVs measured on TEM images in panel H. Unpaired two-tailed *t*-tests were used. Differences were considered significant at ** *p* < 0.01, *** *p* < 0.001. (**J**) Representative TEM images of Calu-3-N NC and DNAJC11-KD cells. Red arrows indicate ER profiles, yellow arrows indicate mitochondria, blue arrows indicate DMV formation. Scale bar: 0.5 μm. (**K**) Average length of ER profiles measured via TEM images shown in (**J**), N > 10 ER profiles. Differences were considered significant at *** *p* < 0.001.

SARS-CoV-2 NSP3 and NSP4 serve as the core constituents necessary for the formation of a pore spanning replication organelle, a structure essential for viral replication. To determine whether DNAJC11 influences the degree of DMV formation induced by overexpressing NSP3 and NSP4, fluorescence imaging was employed to analyze DMV formation in cells overexpressing DNAJC11. Results revealed that ectopic expression of DNAJC11 promoted DMV formation (Figure 6A,B). Western blot analysis confirmed that DNAJC11 overexpression led to an up-regulation of NSP3 expression (Figure S6A). To further investigate the role of DNAJC11 mutants in DMV formation, immunofluorescence assays were performed. Results indicated that deletion of the N-terminal aa 1 to 200 of DNAJC11 had a significant inhibitory effect on NSP3-NSP4-mediated DMV formation (Figure 6C,D). These findings indicate that the J domain of DNAJC11 plays a critical role in facilitating the formation of NSP3-NSP4-mediated DMVs.

### 3.6. DNAJC11 Depletion Impairs SARS-CoV-2-Induced DMV Formation and Disrupts ER and Mitochondrial Morphology

Depletion of DNAJC11 significantly inhibited DMV formation induced by NSP3 and NSP4 overexpression, as demonstrated by immunofluorescence assays. In DNAJC11-KD cells, the number of DMVs was markedly reduced compared to NC cells. Nevertheless, reintroducing DNAJC11 into DNAJC11-KD cells restored DMV formation, confirming the critical role of DNAJC11 in this process (Figure 6E). Western blot analysis confirmed that NSP3 expression was reduced in DNAJC11-KD cells but restored following the reintroduction of DNAJC11 expression (Figure S6B).

Electron microscopy provided further insights, revealing significant reductions in both the quantity and size of DMVs (blue arrows) in DNAJC11-KD cells compared to NC cells; notably, reintroduction of exogenous DNAJC11 in DNAJC11-KD cells restored DMV formation to levels comparable to controls (Figure 6F,G). Furthermore, the ER profile length (red arrow) was significantly shorter in DNAJC11-KD cells than in control cells (Figure 6F,G). To ascertain the impact of DNAJC11 on SARS-CoV-2-induced DMVs, NC and DNAJC11-KD cells were either mock-infected or infected with SARS-CoV-2-VLPs for 48 h. Subsequent electron microscopy revealed a marked reduction in the number and size of DMVs induced by SARS-CoV-2-VLPs.

In SARS-CoV-2-VLP-infected cells, DNAJC11 knockdown significantly reduced the number and size of DMVs, while exogenous DNAJC11 expression in DNAJC11-KD cells rescued both DMV formation and dimensions (Figure 6H,I). To further understand the role of DNACJ11 in ER morphology and SARS-CoV-2-induced DMVs, the length of ER profiles was assessed in DNAJC11-KD cells compared to NC cells after 48 h of SARS-CoV-2-VLP infection. A consistent reduction in ER profile length was observed in DNAJC11-KD cells, as determined by electron microscopy (Figure 6J,K), suggesting compromised ER structural stability and functionality. This disruption may contribute to an unfolded protein response, ER stress, and ER-associated degradation, highlighting the critical role of DNAJC11 in preserving ER integrity during viral infection.

DNAJC11, known as a mitochondrial protein, is also pivotal for mitochondrial structure and dynamics. Transmission electron microscopy revealed significant abnormalities in mitochondrial morphology in DNAJC11-KD cells (white arrow), including vacuolization, elongation, and fusion (Figure 6J). After 48 h of infection with SARS-CoV-2-VLPs, mitochondrial damage was markedly exacerbated in DNAJC11-KD cells compared to mock-treated controls (Figure 6J). These findings suggest that DNAJC11 is a key regulator of mitochondrial and ER morphology and DMV formation during SARS-CoV-2 infection.

### 3.7. DNAJC11 Stabilizes NSP3 Expression to Promote NSP3-NSP4 Interactions

**Figure 7:**
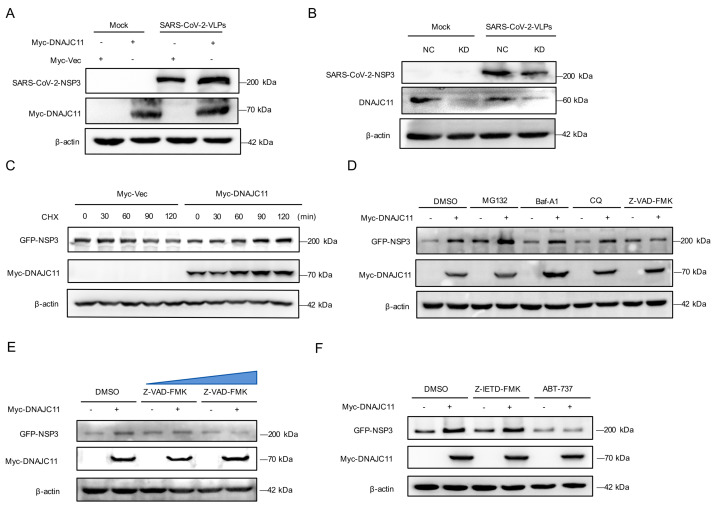
DNAJC11 stabilizes NSP3 expression via endogenous apoptosis pathways. (**A**) Calu-3-N cells were transfected with vector or Myc-DNAJC11 for 24 h, then infected with SARS-CoV-2-VLPs for 24 h. Protein expression levels of NSP3 and Myc-DNAJC11 were quantified using Western blot. (**B**) DNAJC11 NC or KD cells were infected with SARS-CoV-2-VLPs for 48 h. Expression levels of NSP3 and DNAJC11 were quantified using Western blot with indicated antibodies. (**C**) Calu-3-N cells were cotransfected with GFP-NSP3, Myc-Vec, or Myc-DNAJC11-expressing plasmids for 24 h, then treated with CHX for 0, 30, 60, 90, and 120 min. Protein expression levels of GFP-NSP3 and Myc-DNAJC11 were detected by Western blot. (**D**) Calu-3-N cells were cotransfected with GFP-NSP3, Myc-Vec, or Myc-DNAJC11-expressing plasmids for 24 h, then treated with DMSO (control), MG132 (10 µM), Baf-A1 (100 nM), CQ (50 µM), or Z-VAD-FMK (20 µM). Protein expression levels of GFP-NSP3 and Myc-DNAJC11 were detected by Western blot. (**E**) Calu-3-N cells were cotransfected with GFP-NSP3, Myc-Vec, or Myc-DNAJC11-expressing plasmids for 24 h in the presence or absence of DMSO (control) or increasing concentrations of Z-VAD-FMK (20, 50 µM). Protein expression levels of GFP-NSP3 and Myc-DNAJC11 were detected by Western blot. The blue triangle indicates an increase in the concentration of Z-VAD-FMK. (**F**) Calu-3-N cells were cotransfected with GFP-NSP3, Myc-Vec, or Myc-DNAJC11-expressing plasmids for 24 h, then treated with DMSO (control), Z-IETD-FMK (50 µM), or ABT-737 (10 µM). Protein expression levels of GFP-NSP3 and Myc-DNAJC11 were detected by Western blot.

Given that DNAJC11 interacts with NSP3 to promote SARS-CoV-2 replication by facilitating DMV formation, the mechanism of DNAJC11 in SARS-CoV-2-induced DMV formation was investigated. Western blot analysis indicated that NSP3 protein levels were moderately increased in DNAJC11-overexpressing cells compared to Vec-expressing cells during SARS-CoV-2-VLP infection (Figure 7A). Conversely, knockdown of DNAJC11 in SARS-CoV-2-infected cells led to a marked reduction in NSP3 and DNAJC11 expression levels, underscoring the dependence of NSP3 stability on DNAJC11 (Figure 7B).

To assess whether DNAJC11 directly stabilizes NSP3, cycloheximide (CHX), a protein synthesis inhibitor, was used to detect the half-life of NSP3 in DNAJC11-overexpressing cells. Results confirmed that overexpression of DNAJC11 stabilized the expression of SARS-CoV-2 NSP3 (Figure 7C). To further investigate the pathway responsible for DNAJC11-mediated stabilization of NSP3, Calu-3 cells were co-transfected with the NSP3 and either an empty vector or DNAJC11-expressing plasmids, followed by treatment with specific inhibitors. Stabilization of NSP3 by DNAJC11 was abolished by the apoptosis inhibitor Z-VAD-FMK but was unaffected by the proteasome inhibitor MG132 or the autophagy inhibitors chloroquine (CQ) and bafilomycin A1 (Baf-A1) (Figure 7D). Dose-dependent analysis confirmed that increasing concentrations of Z-VAD-FMK progressively suppressed DNAJC11-mediated NSP3 stabilization (Figure 7E). To determine whether this regulation occurred through exogenous or endogenous apoptotic pathways, cells were pretreated with Z-IETD-FMK (exogenous) and ABT-737 (endogenous). Western blotting revealed that only ABT-737 significantly reduced NSP3 protein levels in DNAJC11-overexpressing cells (Figure 7F). To investigate the divergent effects of DNAJC11 on SARS-CoV-2 and HCoV-229E replication, the interaction between DNAJC11 and HCoV-229E NSP3 was examined. Co-IP analysis indicated no detectable binding between DNAJC11 and HCoV-229E NSP3 (Figure S5A). Western blotting showed that DNAJC11 overexpression did not alter HCoV-229E NSP3 protein levels relative to vector controls (Figure S5B). The above results suggest that the negative regulation of HCoV-229E replication by DNAJC11 may not involve its viral NSP3 proteins, which are different with SARS-CoV-2 infection. The working concentrations of all inhibitors were evaluated for potential cytotoxicity using the CCK-8 assay prior to functional experiments (Figure S2E).

**Figure 8:**
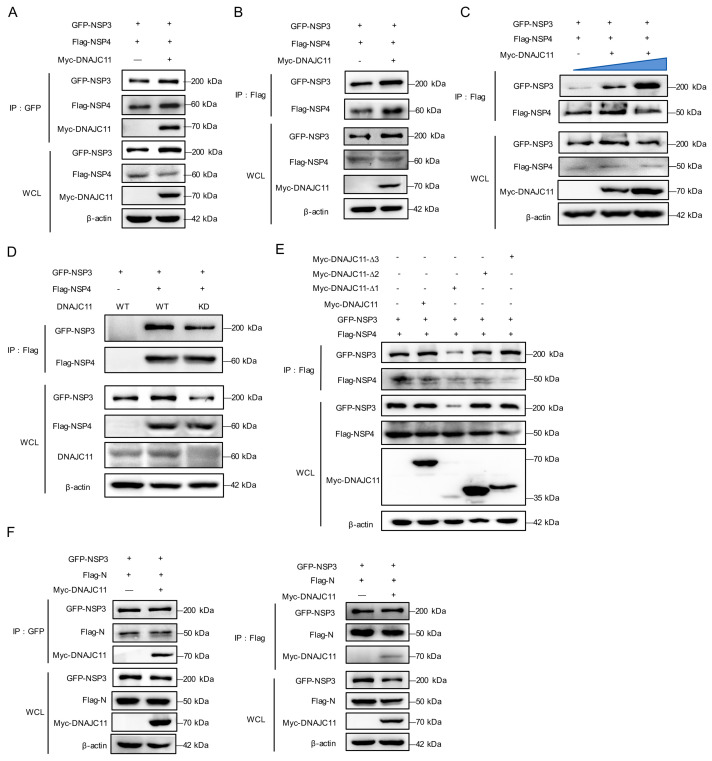
DNAJC11 promotes NSP3-NSP4 interaction. (**A**,**B**) HEK-293T cells were cotransfected with GFP-NSP3, Flag-NSP4, and empty vector or Myc-DNAJC11-expressing plasmids for 24 h. Cell lysates were immunoprecipitated with anti-GFP (**A**) or anti-Flag (**B**) antibodies. WCLs and IP complexes were then analyzed by Western blot with indicated antibodies. (**C**) HEK-293T cells were cotransfected with GFP-NSP3, Flag-NSP4, or plasmids expressing increasing amounts of Myc-DNAJC11 for 24 h. Cell lysates were immunoprecipitated with anti-Flag antibodies. WCLs and IP complexes were then analyzed by Western blot with indicated antibodies. The blue triangle indicates the increasing amounts of Myc-DNAJC11 (0ug, 0.5µg, 1µg). (**D**) DNAJC11 NC or KD cells were cotransfected with GFP-NSP3 or Flag-NSP4 for 24 h. Cell lysates were immunoprecipitated with anti-Flag antibodies. WCLs and IP complexes were then analyzed by Western blot. (**E**) DNAJC11 NC or KD cells were cotransfected with GFP-NSP3, Flag-NSP4, and empty vector or Myc-DNAJC11 or Myc-tagged truncated DNAJC11 mutant-expressing plasmids. Cell lysates were immunoprecipitated with anti-Flag antibodies. WCLs and IP complexes were then analyzed by Western blot. (**F**) HEK-293T cells were cotransfected with GFP-NSP3, Flag-N, and empty vector or Myc-DNAJC11-expressing plasmids for 24 h. Cell lysates were immunoprecipitated with anti-GFP or anti-Flag antibodies. WCLs and IP complexes were then analyzed by Western blot with indicated antibodies.

The interaction between NSP3 and NSP4, mediated by their luminal loops, is critical for initiating host cell membrane rearrangements and DMV formation. Furthermore, interaction between NSP3 and NSP4 in the absence or presence of DNAJC11 was evaluated and compared. Results revealed that DNAJC11 overexpression significantly enhanced the NSP3-NSP4 interaction, irrespective of whether NSP3 or NSP4 was used as the bait protein in the co-IP assays (Figure 8A,B), and a dose-dependent analysis further demonstrated that increasing DNAJC11 levels progressively strengthened this interaction (Figure 8C). In contrast, co-IP assays demonstrated a marked reduction in the NSP3-NSP4 interaction in DNAJC11-KD cells (Figure 8D).

To delineate the specific domains of DNAJC11 involved in promoting the NSP3-NSP4 interaction, various DNAJC11 truncation mutants were analyzed (Figure 5). Co-IP assays revealed that the deletion of residues 1–200 within DNAJC11 resulted in the complete abrogation of the interaction between NSP3 and NSP4, whereas deletions spanning residues 200–400 or 400–560 had no discernible effect (Figure 8E). In addition to NSP3, DNAJC11 was also found to interact with the SARS-CoV-2 N protein (Figure S4). Given the critical role of the binding interaction between SARS-CoV-2 NSP3 and N, co-IP assays were conducted to assess whether DNAJC11 modulates this binding. Results indicated that DNAJC11 did not affect the interaction between NSP3 and the N protein (Figure 8F). Together, these findings indicate that the J domain of DNAJC11 stabilizes the expression of SARS-CoV-2 NSP3, thereby facilitating the interaction between NSP3 and NSP4.

## 4. Discussion

SARS-CoV, MERS-CoV, and SARS-CoV-2 infections are associated with severe, life-threatening respiratory pathologies and lung injuries, leading to high mortality and morbidity in affected individuals [22]. These highly pathogenic CoVs stand in stark contrast to other human CoVs, such as HCoV-NL63, HCoV-229E, and HCoV-OC43, which typically cause mild seasonal respiratory tract infections and are generally considered relatively harmless [2]. CoVs are distinct among RNA viruses due to their exceptionally large genomes and complex strategies for genome expression and regulation [23]. Despite extensive research, the mechanisms underlying their infectivity and pathogenicity remain incompletely understood [24], leaving critical gaps in the development of targeted antiviral therapies. Identifying key host factors that facilitate CoV replication and pathogenesis is crucial for advancing our understanding of these viruses. In this study, we performed the AP-MS to screen the crucial host factors involved in the process of SARS-CoV-2 DMV formation. Among these, the TRAM1, a host transmembrane protein, as critical interacting partners of SARS-CoV-2 NSP3, has been proved to promote replication organelle biogenesis and viral replication [18]. The host fragile X–related (FXR) family proteins FXR1 promotes SARS-CoV-2 replication organelle clustering by interacting with NSP3 and recruiting host translation machinery to enhance viral protein synthesis and replication efficiency [25,26]. We also found the known RTN4, an ER membrane-modulating protein, directly binds SARS-CoV-2 NSP3 and NSP4 to promote replication organelle assembly [27].

DNAJC11, a member of the J protein family, functions as a cofactor of the Hsp40 family [28]. Proteins within this family exhibit highly conserved J domains and are involved in protein translation, folding, and quality control [29]. In recent years, Hsp40 proteins have received considerable attention for their dual roles in maintaining cellular homeostasis and modulating viral pathogenicity [30]. Notably, DnaJ heat Hsp40 member B6 (DNAJB6) has been shown to colocalize and interact with the NS3 protein of Japanese encephalitis virus (JEV), exerting a negative regulatory effect on viral replication [31]. Similarly, Hsp40/DnaJB1 facilitates the nuclear import of influenza A virus (IAV) viral ribonucleoproteins, which are crucial for efficient IAV replication [32]. Furthermore, the Hsp70 protein has been shown to interact with the hexon protein of fowl adenovirus serotype 4 (FAdV-4) in a DNAJC7 protein-dependent manner, significantly reducing FAdV-4 replication [33]. These findings underscore the pivotal roles of Hsp40 proteins, including DNAJC11, in both supporting and regulating viral replication processes.

The novel CoV, SARS-CoV-2, has emerged a primary focus of medical and virological research due to its profound impact on global public health. Understanding the pathogenic mechanisms underlying this disease, particularly the complex virus–host interactions triggered during infection, is essential for developing effective therapeutic strategies. The SARS-CoV-2 genome, one of the largest among RNA viruses, encodes 16 nonstructural proteins, 4 structural proteins, and 9 accessory proteins [34]. Among these, NSP3 is the largest and most functionally diverse, playing pivotal roles in multiple stages of the viral lifecycle [35]. The NSP3 protein of CoV exhibits significant differences in sequence and functional domains, reflecting the evolutionary adaptability and host interaction strategies of different coronaviruses. SARS-CoV-2 and SARS-CoV belong to the subgenus *Sarbecovirus* within the genus *Betacoronavirus*, sharing a relatively close evolutionary relationship. Their NSP3 proteins exhibit approximately 76–85% sequence homology. In contrast, MERS-CoV (subgenus *Merbecovirus*) shows only 45–50% sequence homology in NSP3 compared to SARS-CoV-2, reflecting a more distant evolutionary divergence.

Comprehensive functional genetic and interactomic analyses have identified many previously unknown pro- and antiviral host factors. In this study, DNAJC11 was identified as a host factor interacting with SARS-CoV-2 NSP3, and its depletion correlated with reduced viral replication—suggesting a potential positive regulatory role (Figure 1). Interestingly, DNAJC11 exerted contrasting effects on the replication of other CoVs. For instance, while DNAJC11 enhanced the replication of SARS-CoV-2, it had an inhibitory effect on low-pathogenicity CoVs, such as HCoV-229E. Moreover, DNAJC11 exerted a negative regulatory effect on the replication of other RNA viruses, including VSV, RSV, and EV-D68. These findings suggest a unique and specific role for DNAJC11 in up-regulating SARS-CoV-2 replication (Figure 2).

The mechanisms by which host genes regulate viral replication are numerous and complex. To determine whether DNAJC11 influences this process through innate immune or inflammatory responses, the expression levels of related genes were analyzed. Overexpression of DNAJC11 did not significantly alter the expression of innate immune response-related genes (IFN-β, IFN-α, OASL, ISG15, ISG54, and ISG56) or inflammatory response-related genes during CoV infection (Figure 3). These observations suggest that DNAJC11 does not regulate SARS-CoV-2 replication via the innate immune pathway. Conversely, DMVs are essential ROs for viral replication and transcription during CoV infection [36]. The interaction between NSP3 and NSP4 is crucial for initiating membrane rearrangements necessary for DMV formation [37]. Our results indicated that DNAJC11 directly interacted with SARS-CoV-2 NSP3 DPUP-Ubl2 domains through its unique J domain (Figure 4 and Figure 5). Furthermore, ectopic expression of DNAJC11 promoted DMV formation and increased DMV numbers, whereas the knockdown of DNAJC11 significantly reduced both the size and number of DMVs (Figure 6). Currently, DMVs are believed to originate from the ER or via alternative mechanisms, including autophagy [38]. In our study, DNAJC11 depletion resulted in pronounced morphological damage to the ER and mitochondria during SARS-CoV-2 infection (Figure 6). DNAJC11 was initially characterized as a constituent of the mitochondrial membrane, where it interacts with key protein complexes governing mitochondrial architecture and biogenesis: specifically, the MICOS complex (mitochondrial contact site and cristae organizing system) and the SAM complex (sorting and assembly machinery) [39]. As the primary energy-producing organelles of the cell, mitochondria are essential for many cellular processes [40]. Since DNAJC11 has known mitochondrial roles, perturbations affecting it might lead to broader mitochondrial or cellular disturbances, resulting in downstream modifications in DMV formation or viral replication. Our findings provide additional evidence of the importance of mitochondrial functional integrity in DMV formation and SARS-CoV-2 replication.

DNAJC11 markedly enhanced the stability of SARS-CoV-2 NSP3, as demonstrated by CHX chase assays, while pharmacological inhibition pinpointed the endogenous apoptotic pathway as a key regulatory axis for this stabilization (Figure 7). Stabilization of NSP3 is critical for its interaction with NSP4, a prerequisite for the formation of SARS-CoV-2 DMVs and a process conserved across several positive-sense single-stranded RNA viruses such as poliovirus and hepatitis C virus [41,42]. To elucidate the regulatory mechanisms underlying SARS-CoV-2 replication, the role of DNAJC11 in facilitating NSP3-NSP4 interactions was investigated. Results demonstrated that DNAJC11 enhanced this interaction by stabilizing NSP3, thereby increasing DMV formation (Figure 8). Although the interaction between NSP3 and the N protein has been associated with increased viral RNA transport and infectivity [43], subsequent co-IP analysis indicated that DNAJC11 did not influence NSP3-N binding (Figure 8).

While this study establishes DNAJC11 as a functional cofactor of SARS-CoV-2 NSP3, there are several mechanistic limitations. The reason that DNAJC11 inhibits other coronaviruses and RNA viruses while enhancing SARS-CoV-2 lacks mechanistic explanation; it remains unclear whether this stems from virus-specific utilization of DMV biogenesis pathways or host factor rewiring. Downstream signaling pathways of ER–mitochondria morphological changes, for example, ER stress responses, and mitochondrial metabolism, also remain uncharacterized. These unresolved mechanisms and issues also represent directions for our future exploration.

**Figure 9:**
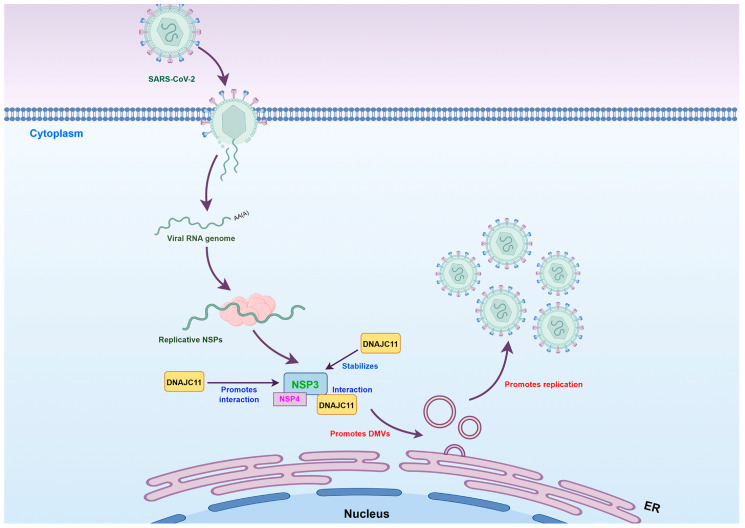
A schematic model of DNAJC11 promoting DMV formation and enhancing SARS-CoV-2 replication. DNAJC11 interacts with SARS-CoV-2 NSP3 to stabilize its expression and promotes NSP3-NSP4 binding, thereby facilitating the formation of DMVs and enhancing viral replication.

This study provides the first evidence identifying DNAJC11 as a novel cofactor that directly interacts with the SARS-CoV-2 NSP3 protein through its J domain. Importantly, DNAJC11 exhibited a distinct functional profile, markedly inhibiting the replication of other CoVs and RNA viruses while specifically enhancing the replication of SARS-CoV-2. These findings highlight a novel mechanism by which DNAJC11 and NSP3 cooperate to drive DMV biogenesis, a critical process for SARS-CoV-2 replication. DNAJC11 was shown to promote DMV formation and induce morphological changes in the ER and mitochondria, essential for supporting viral replication (Figure 9). These findings offer valuable insights into the role of host cofactors in SARS-CoV-2 replication, the functional significance of DNAJC11, potential therapeutic targets for anti-SARS-CoV-2 strategies, and the mechanisms underlying DMV formation during the replication of positive-strand RNA viruses.

## Data Availability

All data generated or analyzed during this study are included in the article and Supplementary Materials. Raw data and microscopy images, materials, and sequence information are available upon request, without undue reservation.

## Supplementary Materials

The following supporting information can be downloaded at: https://www.mdpi.com/article/10.3390/v17081025/s1, Table S1: Primes designed for qRT-PCR; Orignal data for WB: Orignal data for Western blot; Figure S1. Large-scale functional and interactome analyses; Figure S2. CCK8 cell viability assay; Figure S3. DNAJC11 positively regulates type I IFN production and ISG expression; Figure S4. DNAJC11 does not interact with other SARS-CoV-2 proteins, except for N; Figure S5. DNAJC11 does not interact with HCoV-229E NSP3, and dose not affect its stability; Figure S6. DNAJC11 positively regulates the expression of SARS-CoV-2 NSP3.
