# Supplementary material for: DNAJ Homolog Subfamily C Member 11 Stabilizes SARS-CoV-2 NSP3 to Promote Double-Membrane Vesicle Formation
Source: Viruses. 2025 Jul 22;17(8):1025. doi: 10.3390/v17081025 (PMC12390733; doi:10.3390/v17081025)
Supplement: Supplementary file 1 [file viruses-17-01025-s001.zip › viruses-3665456 -Supplementary Figures.pdf]

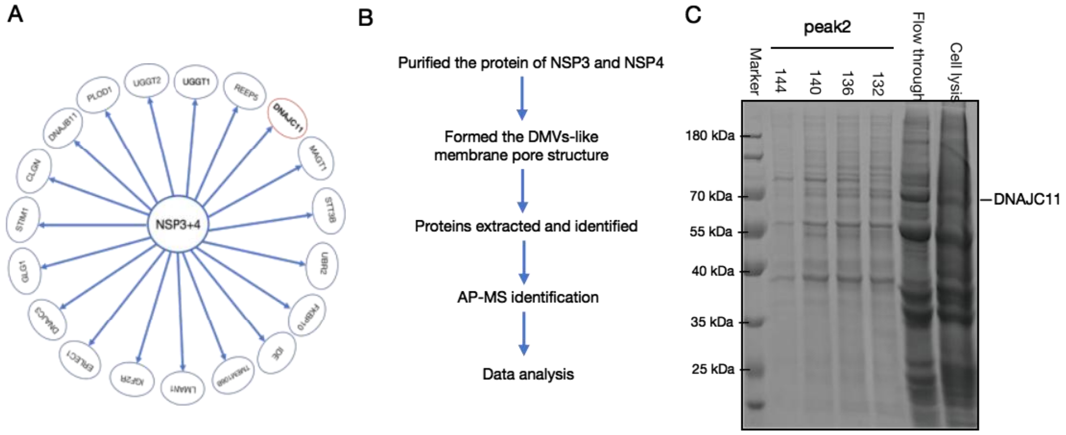

Figure S1. Large-scale functional and interactome analyses. (A) Graph depicts high-confidence host-binding proteins for SARS-CoV-2 NSP3 and NSP4 (blue rectangles) according to literature review. (B) Schematic of AP-MS sample preparation workflow. (C) Western blot analysis of purified NSP3-NSP4 proteins complex showing a ~60 kDa protein, identified as DNAJC11.

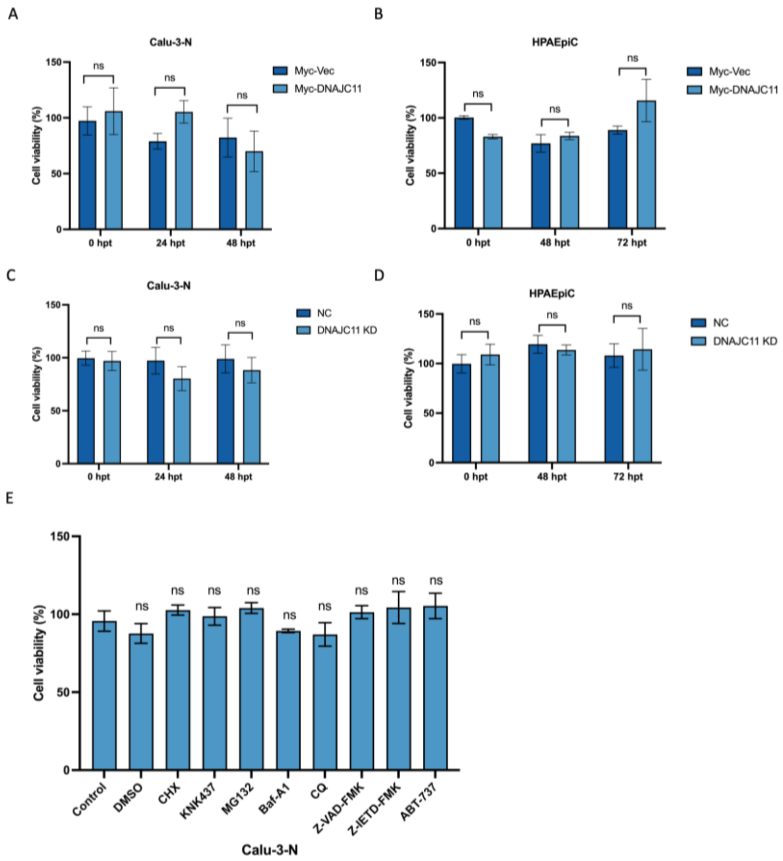

Figure S2. CCK8 cell viability assay. (A, B) Calu-3-N (A) and HPAEpiC cells (B) were transfected with Myc-Vec or Myc-DNAJC11 expressing plasmids for indicated times, and the cell viabilities were evaluated using CCK8 kit. (C, D) Calu-3-N (C) and HPAEpiC cells (D) were transfected with NC or DNAJC11 KD for indicated times, and the cell viabilities were evaluated using CCK8 kit. (E) Calu-3-N cells were treated with different concentrations of the drugs, and the drug's cytotoxicity was evaluated by CCK8 kit. Data are presented as mean ± SD of three independent experiments (n=3). Statistical significance was analyzed by student's *t*-test (ns represents no significant).

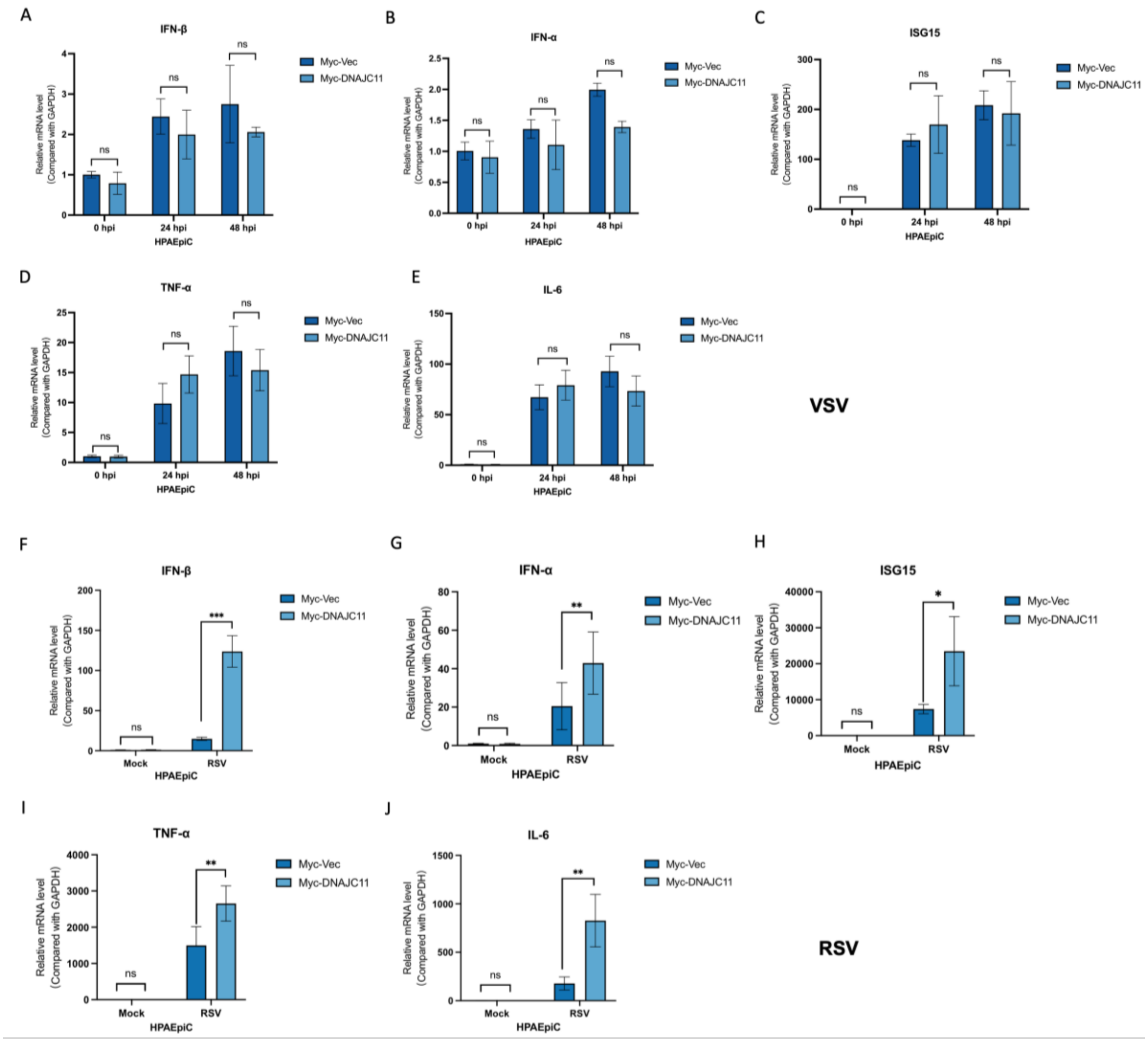

Figure S3. DNAJC11 positively regulates type I IFN production and ISG expression. (A-E) HPAEpiCs were transfected with vector or Myc-DNAJC11 for 24 h, then infected with VSV for 24 h and 48 h. mRNA levels of IFN-β (A), IFN-α (B), ISG15 (C), TNF-α (D), and IL-6 (E) were quantified using qRT-PCR. (F-J) HPAEpiCs were transfected with vector or Myc-DNAJC11 for 24 h, then infected with RSV A2 strain for 24 h. mRNA levels of IFN-β (F), IFN-α (G), ISG15 (H), TNF-α (I), and IL-6 (J) were quantified using qRT-PCR.

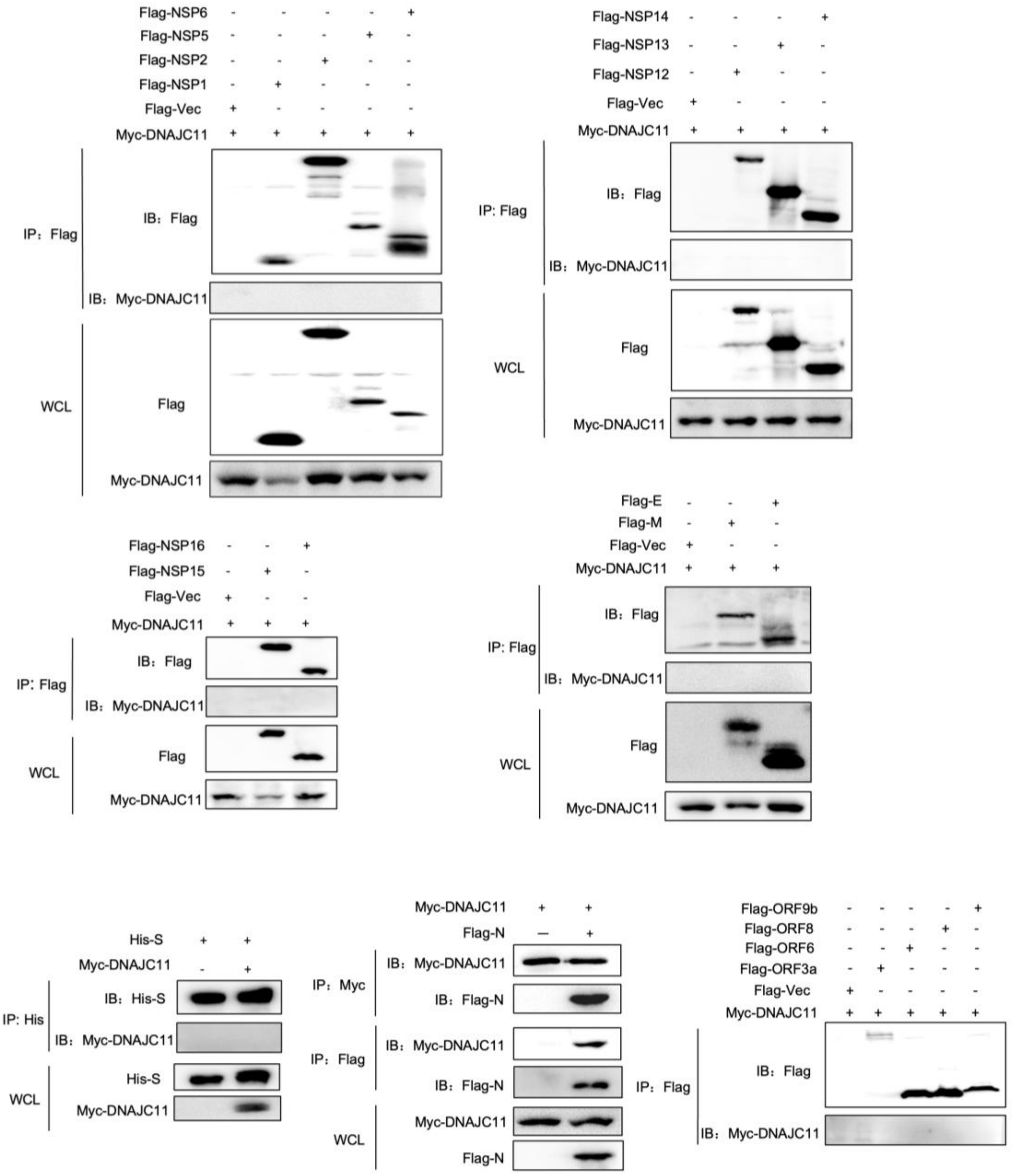

Figure S4. DNAJC11 does not interact with other SARS-CoV-2 proteins, except for N. HEK293T cells were cotransfected with vector or SARS-CoV-2 viral protein (NSP1, NSP2, NSP5, NSP6, NSP12, NSP13, NSP14, NSP15, NSP16, E, M, S, N, ORF3a, ORF6, ORF8, ORF9b) and Myc-DNAJC11 for 24 h. Cells were lysed and immunoprecipitated with indicated antibodies. WCL and IP complexes were analyzed by western blotting.

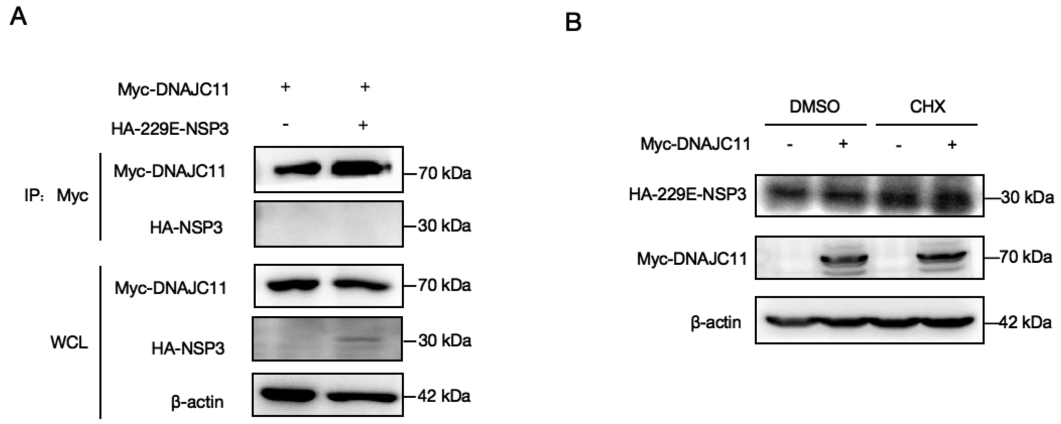

Figure S5. DNAJC11 does not interact with HCoV-229E NSP3, and dose not affect its stability. (A) HEK293T cells were cotransfected with vector or HA-NSP3 and Myc-DNAJC11 for 24 h. Cells were lysed and immunoprecipitated with anti-Myc antibodies. WCLs and IP complexes were analyzed by western blotting. (B) Calu-3 cells were cotransfected with HA-229E-NSP3, Myc-Vec, or Myc-DNAJC11 plasmids for 24 h, then treated with CHX for 120 min. Protein expression levels of HA-NSP3 and Myc-DNAJC11 were detected by western blotting.

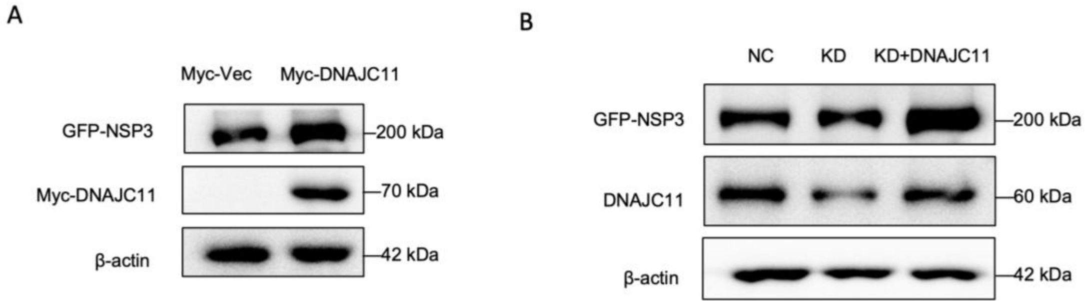

Figure S6. DNAJC11 positively regulates the expression of SARS-CoV-2 NSP3. (A) Calu-3 cells were cotransfected with GFP-NSP3, mCherry-NSP4, and empty vector or Myc-DNAJC11 plasmids for 24 h. The expression of GFP-NSP3 and Myc-DNAJC11 were detected by western blot. (B) DNAJC11 NC-, KD-, or KD-supplemented DNAJC11 cells were cotransfected with GFP-NSP3 or mCherry-NSP4 for 48 h. The expression of GFP-NSP3 and Myc-DNAJC11 were detected by western blotting.
